# Calr-mediated calcium buffering: a molecular barrier to in vivo cardiac reprogramming

**DOI:** 10.1038/s41392-026-02901-3

**Published:** 2026-08-07

**Authors:** Johnny Kim, Hans R. Schöler, Kee-Pyo Kim

**Affiliations:** 1 The Department of Cardiovascular Therapeutics at TRON, Translational Oncology at the University Medical Center of the Johannes Gutenberg University Mainz gGmbH, Mainz, Germany; 2 Vascular Biology and Therapeutics Program and Department of Comparative Medicine, Yale University School of Medicine, New Haven, CT USA; 3 Department of Cell and Developmental Biology, Max Planck Institute for Molecular Biomedicine, Münster, Germany; 4 Department of Medical Life Sciences, College of Medicine, The Catholic University of Korea, Seoul, Republic of Korea; 5 Department of Medical Sciences (Graduate School), College of Medicine, The Catholic University of Korea, Seoul, Republic of Korea

**Keywords:** Reprogramming, Transdifferentiation

In a recent publication in *Cell Stem Cell*, Cai et al.^1^ employed an in vivo Perturb-seq strategy to systematically identify molecular barriers that limit direct cardiac reprogramming following myocardial infarction (MI). This study provides a conceptual and technological framework for improving fibroblast-to-cardiomyocyte conversion in vivo and identifies a key regulatory pathway that can be targeted to enhance cardiac regeneration.

The limited regenerative capacity of the adult mammalian heart and the resulting loss of functional myocardium after MI underscore the urgent need for strategies that can restore cardiac muscle and improve heart function. Direct cardiac reprogramming has emerged as a promising approach for myocardial repair whereby fibroblasts can be directly converted into induced cardiomyocytes (iCMs). Pioneering studies have demonstrated that defined transcription factors including Mef2c, Gata4, and Tbx5 (collectively referred to as MGT), and later extended combinations such as Myocd and Sall4 (collectively termed MGTMyoS) can induce transdifferentiation of fibroblasts to a cardiomyocyte-like state both in vitro and in vivo.^2–4^ However, the efficiency and fidelity of in vivo direct reprogramming remain low, representing a major obstacle for clinical translation. This limitation is thought to arise, at least in part, from the complex post-injury microenvironment, where inflammatory signaling, extracellular matrix remodeling, and cellular stress responses collectively constrain cell fate conversion. Despite growing recognition of these influences, the molecular mechanisms that restrict cardiac reprogramming efficiency in the injured heart have not been defined. A comprehensive and quantitative understanding of the molecular barriers operating in this context is therefore needed to guide the development of more effective cardiac reprogramming strategies.

To address this gap, the authors developed a systematic in vivo Perturb-seq platform that combines short hairpin RNA (shRNA)-based loss-of-function screening with single-cell transcriptome analysis. Importantly, rather than relying on direct delivery of MGTMyoS and shRNA viruses to the injured heart tissue, the authors transplanted fibroblasts pre-transduced with doxycycline-inducible MGTMyoS and constitutively expressed shRNAs into the injured heart. This approach established a uniform starting population of cells and enabled systematic evaluation of genetic perturbations during in vivo cardiac reprogramming within a physiologically relevant environment. The authors systematically targeted 140 candidate genes (131 putative reprogramming barrier genes annotated as transcription factors, epigenetic modifiers, kinases, and receptors along with 9 previously known regulators) and used single-cell transcriptome analysis to quantify how each perturbation influences reprogramming outcomes, enabling precise assessment of gene-specific effects and cell-to-cell variability.

**Fig. 1:**
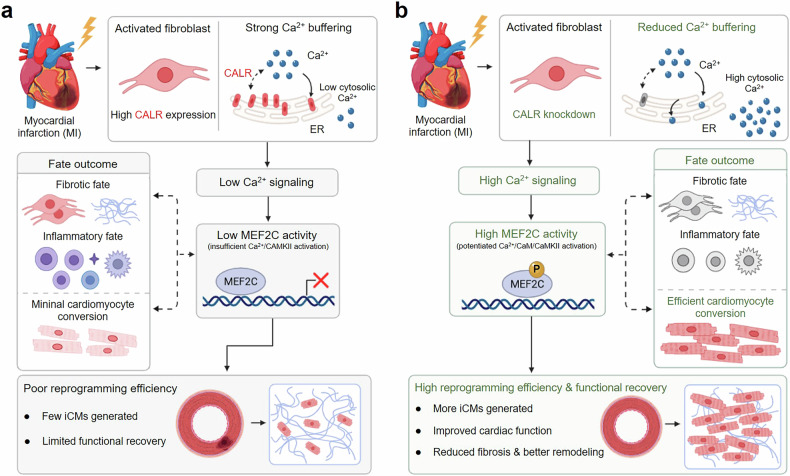
Attenuation of Calr-mediated calcium buffering enhances direct cardiac reprogramming. **a** Following myocardial infarction (MI), activated cardiac fibroblasts exhibit elevated calreticulin (CALR) expression, which enhances endoplasmic reticulum (ER) calcium (Ca²⁺) buffering and reduces cytosolic Ca²⁺ availability. This results in insufficient activation of the Ca²⁺/calmodulin (CaM)/CaMKIIδ signaling pathway, leading to reduced MEF2C transcriptional activity and limited induction of cardiogenic gene programs. Consequently, fibroblasts predominantly adopt fibrotic and inflammatory fates, resulting in poor cardiomyocyte conversion and limited functional recovery. **b** In contrast, suppression of CALR relieves pathological calcium buffering, increasing cytosolic Ca²⁺ levels and restoring Ca²⁺-dependent signaling. Elevated Ca²⁺ activates the CaM–CaMKIIδ cascade, which enhances MEF2C transcriptional activity and promotes cardiogenic gene expression. This shift redirects fibroblast fate toward cardiomyocyte lineage, resulting in increased generation of induced cardiomyocytes (iCMs), with improved structural maturation, reduced fibrosis, and enhanced cardiac function. Generated with BioRender.com

Among the identified candidates, the calcium regulator Calreticulin (Calr) emerged as a top-ranked barrier to direct cardiac reprogramming. Calr expression was markedly upregulated in fibroblasts within the infarcted heart, suggesting a key role in restricting cell fate conversion during cardiac repair and highlighting it as a context-dependent barrier in the injured microenvironment (Fig. 1). Functional validation revealed that Calr knockdown markedly enhanced cardiac reprogramming across multiple experimental settings. In vitro, Calr depletion increased the generation of α-Actinin⁺/cTnI⁺ iCMs and promoted their functional maturation, as evidenced by enhanced cardiac gene expression, organized sarcomere formation, spontaneous beating, and synchronized calcium oscillations. Importantly, these effects were conserved in human iPSC-derived cardiac fibroblasts and primary fibroblasts isolated from myocardial infarction patients, highlighting the translational potential of targeting Calr to improve cardiac reprogramming. In vivo, Calr suppression significantly increased fibroblast-to-cardiomyocyte conversion within the infarcted heart and generated structurally mature iCMs with rod-shaped morphology and organized sarcomeres. Moreover, combining Calr knockdown with tdMGT improved cardiac function, enhanced ventricular synchrony, and attenuated fibrotic remodeling following myocardial infarction, demonstrating that removal of this molecular barrier translates into clinically meaningful cardiac repair.

Mechanistically, the authors demonstrate that Calr depletion increases cytosolic Ca²⁺ levels, thereby activating a calmodulin (CaM)–CaMKIIδ signaling cascade that enhances MEF2C transcriptional activity (Fig. 1). Notably, this effect was not driven by increased MEF2C expression, but rather by modulation of its activity, highlighting a post-translational regulatory mechanism. Pharmacological elevation of intracellular Ca²⁺ recapitulated the effects of Calr knockdown, including enhanced reprogramming outcomes. Importantly, Calr knockdown partially bypassed the requirement for exogenous Mef2c, suggesting that restoration of endogenous signaling networks can compensate for the absence of an extrinsic transcriptional input. These findings establish Calr-mediated calcium buffering as a central gatekeeper of cardiac fate plasticity.

Despite these advances, several important questions remain. First, the extent to which multiple identified barriers act synergistically or redundantly remains unclear, and it will be exciting to see if combinatorial perturbation strategies could further enhance reprogramming efficiency. Second, the long-term functional stability, electrophysiological coupling with existing myocardium, and overall safety of iCMs require further investigation. Along this line, the question arises to what extent iCMs resemble the properties of native cardiomyocytes. Third, although immune-mediated barriers to cardiac reprogramming have begun to be defined,^5^ further elucidation of the cellular and molecular immune circuitry regulating cardiac fate conversion will be important. Fourth, translation to large animal models and human in vivo systems will be essential to validate and advance the clinical applicability of these findings. Finally, it will be important to define the therapeutic window for transdifferentiation of cardiac fibroblasts after MI, specifically whether and when reprogramming remains feasible, including at later stages when fibrosis is established and cardiac function is already compromised.

In conclusion, the study by Cai et al.^1^ establishes in vivo Perturb-seq as a powerful platform for decoding the molecular constraints of in vivo cardiac reprogramming and identifies Calr-mediated calcium signaling as a key pathological barrier to cardiac regeneration. By shifting the field from descriptive candidate-based studies to global, quantitative, and systematic dissection, this work provides a foundation for the rational design of more effective cardiac regenerative strategies. In the future, targeted delivery strategies employing adeno-associated virus (AAV) vectors, lipid nanoparticle (LNP)-based mRNA systems, and/or small-molecule approaches may enable localized and tightly controlled modulation of reprogramming factors and Calr-associated signaling pathways. Applied either alone or in combination, these interventions may enhance the safety, controllability, and therapeutic efficacy of cardiac regeneration.
